# A Treatise on the Eclectic Southern Practice of Medicine

**Published:** 1854-04

**Authors:** 


### Art. VI.—A TREATISE ON THE ECLECTIC SOUTHERN PRACTICE OF MEDICINE.

By J. Cam. Massie, M. D. Philadelphia; Thomas Cowperthwaite & Co.; pp. 716. 1854.

When we saw it announced, some time ago, that Dr. Massie, of Texas, was engaged in the preparation of a work on "Southern Practice," we felt not a little concerned for the character of the profession. We had seen some reports of cases from the pen of this gentleman in the *New Orleans Journal of Medicine*, and had otherwise previously obtained some knowledge of his fitness for such a task. We confess, that the idea of his putting himself forward as the exponent and representative of *Southern Medicine*, was to us a most unwelcome one. We could see in the project nothing but evil to the profession and to the public. We knew that the experience, the extended observation, the reading, and habits of mental application necessary to the execution of such a work in a useful and creditable manner, were all wanting in this writer. We expected, if he carried out his design, to see a book, made up more with the scissors than the pen, abounding in all sorts of crudities and absurdities, full of old cumbrous recipes and old exploded notions. In no respect has he disappointed us save one, and that is in making his book a good deal worse than we expected it to be. We cannot say that in regard to medical science it falls below our expectations, utterly destitute as it is of all pretensions of that sort; but the author has imposed upon it a gratuitous degradation in making its morality as bad as its science. But he has been honest in one thing; he calls his production by the right name—the "*Eclectic* Southern Practice." We rejoice that it is in a work styled "*Eclectic*," and not in a work emanating from scientific medicine, that regular *dram-drinking* is inculcated, and the young men and old men of the South are instructed in the method of committing fornication without the risk of contracting the diseases incident to promiscuous sexual intercourse. We thank Dr. Massie for calling his book by its true title, which releases us from all obligation to bestow upon it any further notice, and, what is of far greater importance, will disarm it, to a great extent, of the power to do mischief. He says he meant to make "a book which would be a valuable aid to every father of a family;" but we dare believe there are few fathers anywhere, whatever their habits, who would be willing that the eyes of their unsophisticated sons and daughters should fall upon some of his obscene pages. We trust that it will find its way into few families, and can see no reason why it should find a resting place upon the shelf of any physician. There is but one proper resting place for such a book—that *limbo of vanity* in which all the bad things and all the foolish things of the press are sure, sooner or later, to be swallowed up.

